# Association between Birth Weight and Mortality over the Two First Months after Birth in Feline Species: Definition of Breed-Specific Thresholds

**DOI:** 10.3390/ani13111822

**Published:** 2023-05-31

**Authors:** Amélie Mugnier, Virginie Gaillard, Sylvie Chastant

**Affiliations:** 1 NeoCare, Université de Toulouse, ENVT, 31300 Toulouse, France; amelie.mugnier@envt.fr (A.M.); sylvie.chastant@envt.fr (S.C.); 2 Royal Canin Research Center, 30470 Aimargues, France

**Keywords:** birth weight, kitten, risk factor, threshold, neonatal mortality, feline

## Abstract

**Simple Summary:**

Low birth weight has been shown to increase the risk of mortality in feline species. Thus, it is important to provide pet professionals with weight thresholds in order to enable them to make the best use of this simple and inexpensive, but essential, management practice, i.e., weighing kittens at birth. Based on data collected from 194 French catteries, this study defined birth weight thresholds which allow for the identification of kittens at higher risk of 0–2 months mortality in 15 breeds (5596 kittens). Two thresholds were identified, classifying kittens into three groups: normal, low, and very low birth weight, characterized by low, moderate, and high risk of 0–2 months mortality, respectively. Values defining very low birth weight kittens varied between 60 g and 78 g depending on the breed and the values defining low birth weight kittens were between 74 g and 104 g. When used as alarm thresholds, these values will facilitate the detection of kittens requiring specific nursing.

**Abstract:**

In many species, low birth weight is identified as a major determinant for neonatal survival. The objectives of the present study were (i) to assess, in a large feline purebred population, the impact of birth weight on 0–2 months mortality in kittens, and (ii) if such mortality occurs, to define cut-off values for birth weight to identify at-risk kittens. Data from 5596 kittens from 15 breeds and provided by 194 French breeders were analysed. A logistic mixed model was used to identify low birth weight, being a male, and being born in a large litter as significant risk factors for kitten mortality during the first two months after birth. Classification and regression tree analysis was used to define the thresholds, first at the species level and, when possible, at the breed level. Two thresholds were defined to group kittens into three categories: low, moderate, or high risk of 0–2 months mortality (normal, low, and very low birth weight, respectively). In our population, 19.7% of the kittens were classified as low birth weight and 1.9% as very low birth weight. Critical thresholds may differ between breeds with similar birth weight distributions and equivalent mortality rates (e.g., Russian Blue/Nebelung vs. Egyptian Mau). These critical birth weight thresholds, established in 15 breeds, could be used to identify kittens requiring more intensive nursing to improve survival.

## 1. Introduction

Kitten mortalities during the first two months after birth impacts the welfare of the animals, affects the emotional state of breeders, and impacts the financial stability of their facilities. Despite its interest for veterinarians, breeders, and pet owners, feline neonatalogy remains poorly explored and neglected [1], especially when considering its potential impact on health during life [2]. This topic seems to be gaining momentum in the scientific community [3,4,5], probably due to the growing interest in cats, especially purebred cats, as companion animals [6]. Adequate management of newborns is crucial for their survival, and the early identification of at-risk kittens is one of the keys to successful breeding [5,7].

In many mammalian species, birth weight has been identified as a major determinant for neonatal survival [8]. The relationship between low birth weight (LBW) and neonatal mortality has been poorly explored in feline species and only in a small population from a single cattery and breed [9]. With birth weight differences of more than 20% between breeds (mean values between 82 g and 118 g for Persian and Maine Coon, respectively [10,11,12]), previous studies on cats suggest the need to work at the breed level and not the species level as a whole.

The first objective of the present study was to assess the impact of birth weight on 0–2 months mortality in kittens in a large feline purebred population with multiple breeds. Since weighing at birth is an easy action to implement in the field, it is necessary to determine decisional birth weight thresholds: in the second part of this work, cut-off values for birth weight to identify at-risk kittens were determined by breed.

## 2. Materials and Methods

### 2.1. Study Population

This study was constructed from the same data collection presented in Mugnier et al. [10]. Briefly, data were collected through a questionnaire administered to French purebred cat breeders from 2016 to 2020 and completed on a voluntary basis. The recorded data used for the present study included information about the litter (date of birth, breed, litter size, and the presence of stillbirths in the litter), queen (identity), and kittens (sex, birth weight, and mortality during the first two months after birth). Varieties of the same feline breed were grouped for the analyses: Abyssinian and Somali, Exotic and Persian, and Russian Blue and Nebelung (including short and long-haired version of each). Finally, Orientals were grouped with Mandarins (long-haired version), Siamese (colorpoint version), and Balinese (long-haired colorpoint version).

From the total database, several exclusion criteria were applied to select the study population for the current study. All stillborn neonates, kittens born before 2000, and/or with no birth weight provided and/or with unknown status regarding mortality at two months of age were excluded. Finally, only kittens from breeds with at least 100 individuals were included in the final dataset.

### 2.2. Data Management and Analysis

All statistical analyses were performed using R software version 4.2.1 [13]. Results with *p*-values less than 0.05 were considered significant. Statistical uncertainty was assessed by calculating 95% binomial confidence intervals (95%CI).

#### 2.2.1. Impact of the Kittens’ Characteristics at Birth on Their Mortality Risk

A logistic mixed model was fitted using the package lme4 [14] to determine factors affecting mortality rate during the first two months after birth (binary outcome variable). The fixed-effects introduced into the models were: birth weight, sex, presence of at least one stillborn in the litter, litter size (total number of kittens born alive), litter weight heterogeneity, and season of birth. The queen was introduced as a random effect to deal with the non-independence of kittens born from the same queen. Litter weight heterogeneity represented within-litter variation of birth weights and was expressed as the coefficient of variation (CV), the ratio of the standard deviation to the mean of kitten birth weights from a given litter [15]. Season of birth was determined using meteorological seasons in Metropolitan France: autumn (September, October, November), spring (March, April, May), summer (June, July, August), and winter (December, January, February). The high number of breeds (n = 15) represented prevented the introduction of breed as a fixed-effect (convergence failure). Breed effect was nevertheless introduced by classifying continuous parameters influenced by breed (birth weight, litter size and litter heterogeneity; all *p* < 0.001, Kruskal–Wallis rank sum test) using breed-specific quartiles. For each breed and each parameter, kittens were divided into four groups based on the calculated quartiles: Q1 for kittens with a value in the lowest 25% of the study population (lower than the first quartile); Q2, with a value between the first quartile and the median; Q3, with a value between the median and the third quartile, and Q4, a value in the highest 25% (higher than the third quartile).

All the explanatory parameters included in the model were thus categorical variables, and, for each of them, the category describing the lowest mortality rate was taken as a reference in the model. Before interpretation, the final model was assessed using the package *performance* [16], and post hoc tests were performed using the *glht* function of the *multcomp* package [17].

#### 2.2.2. Birth Weight Thresholds

Classification and regression tree (CART) analysis was used to identify kittens at increased risk of mortality during their first two months after birth. This nonlinear and nonparametric model based on the recursive partitioning method consists of repeatedly partitioning the data into several subgroups, so that the results in each final subgroup are as homogeneous as possible [18,19]. The method provides rules (here, cut-off value) used for predicting the outcome variable (here, status dead or alive at 2 months). The Gini index was used as the splitting method, and a 10-fold cross-validation repeated 5 times was used as the method for testing the trees obtained. The Root Mean Squared Error (RMSE) was used to select the optimal model using the smallest value.

Analyses were performed using the R packages *rpart* [20] and *caret* [21]. The procedure was first conducted in the total study population, i.e., at the feline species level, and then separately for each breed, i.e., at the breed level.

## 3. Results

### 3.1. Population Characteristics

**Figure 1:**
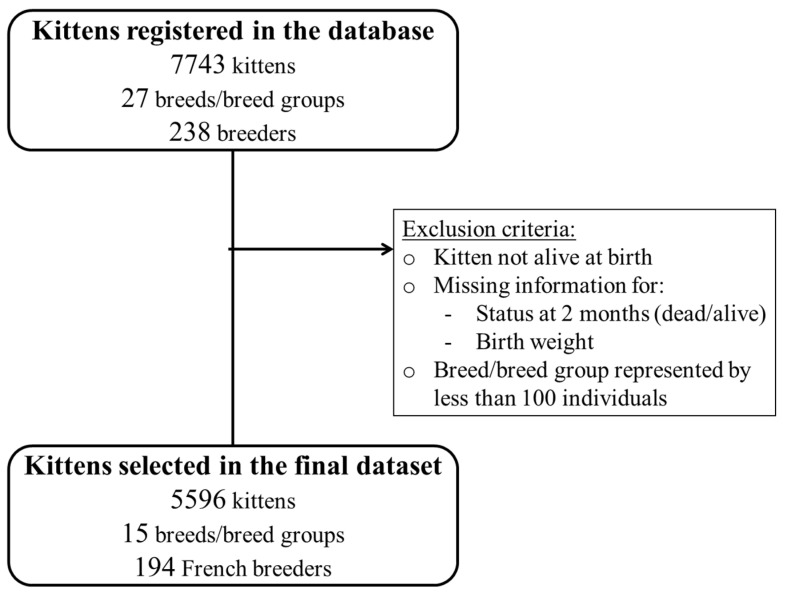
Data selection process. Varieties of the same feline breed were grouped for the analyses (as described in Mugnier et al. [10]).

Data from a total of 5596 live-born kittens from 15 breeds, 1507 litters, and 194 French catteries were included in this study (Figure 1). The description of the population is presented in Table 1. Litters were born between 2000 and 2020 with 75% of the litters born after 2010. The number of kittens included per breed ranged from 108 for Russian Blue/Nebelung to 892 for Maine Coon (median = 274). In 83% of litters, no stillbirths were reported. Sex ratio was calculated at 1.1 (2801 males vs. 2459 females); 68% of the kittens included were born in spring or summer (3816/5596). Birth weights ranged from 36 g (a Persian/Exotic kitten) to 182 g (a Norwegian Forest Cat kitten) with a mean of 101.9 g (SD = 19.4). Average birth weights per breed ranged from 85.5 g (SD = 15) for Persian/Exotic to 119 g (SD = 18.7) for Maine Coon. The global mean litter size at birth was 3.9 (SD = 1.6) kittens and varied at the breed level from 3.1 (SD = 1.1) for Persian/Exotic to 4.4 (SD = 1.7) for Russian Blue/Nebelung. The global median litter heterogeneity was 8.1% (IQR: 5.3–11.6) and varied at the breed level from 6% (IQR: 4.7–9.4) for Russian Blue/Nebelung to 10.2% (IQR: 6.4–14.4) for Bengal.

### 3.2. Identification of Neonatal Mortality Risk Factors

**Figure 2:**
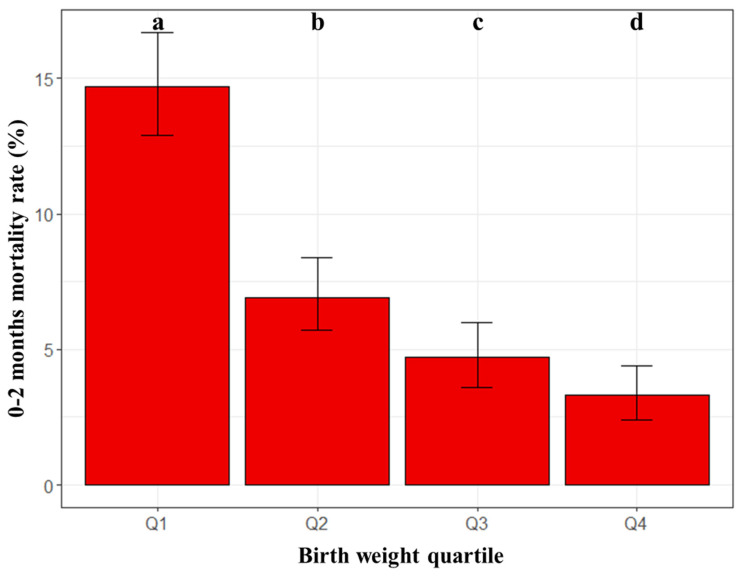
Mortality rates over the first two months of life by birth weight quartiles (n = 5596 kittens). Birth weight categories were constructed based on quartile values calculated at the breed level. The error bars represent statistical uncertainty (95% binomial confidence intervals). Mortality rates were significantly different between the four groups (Tukey post hoc test after the generalized linear mixed-effects model; different letters at the top of the bars indicate significant differences).

A total of 7.6%, 95% CI [6.9, 8.3], live-born kittens died over the first two months after birth. Results of mixed effects logistic regression are shown in Table 2. Mortality rates over the first two months were significantly different between all birth weight quartiles, and they increased when birth weights decreased (Figure 2). In addition, the mortality rate was statistically significantly higher in male kittens compared with females, but with a negligible biological difference (6.7%, 95% CI [5.8, 7.7] vs. 6.6%, 95% CI [5.6, 7.6]; *p* = 0.001), and it was higher for kittens born in summer compared with those born in spring (9.7%, 95%CI [8.4, 11.2] vs. 5.9%, 95%CI [4.9, 7]; *p* = 0.015). Finally, the lowest mortality rate was observed in Q2-sized litters, without difference with Q1 or Q3-sized litters but with a significant increase in Q4-sized litters (4.1%, 95%CI [2.9, 5.4] vs. 11.5%, 95%CI [9.6, 13.7]; Table 2). The variance of the random effect parameter, i.e., the queen, was 3.29 (SD = 1.7). The model’s total explanatory power was 0.54 (conditional R2) and the part related to the fixed effects alone (marginal R2) was 0.14.

### 3.3. Birth Weight Cut-Off Values

**Figure 3:**
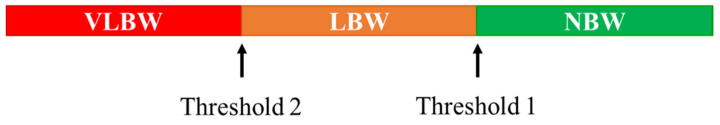
Classification of kittens according to their birth weight. Thresholds were determined by the CART method. VLBW: very low birth weight; LBW: low birth weight; NBW: normal birth weight.

Cut-off values for birth weight regarding the 0–2 months mortality rate were first identified for the species, then, in the second step, refinement was sought by breed. At the species level, two thresholds, 82 and 60 g, were identified by CART analysis. Kittens were thus divided into three groups depending on their mortality risk (Figure 3): normal birth weight (NBW, kittens with birth weight at or above the Threshold 1) with the lowest mortality rate, low birth weight (LBW, between the two thresholds) with intermediate mortality rate and very low birth weight (VLBW, under the Threshold 2) with the highest mortality rate.

In the absence of breed-specific threshold, values identified at the species level were attributed. Table 3 presents threshold birth weight values for each breed/breed group. Depending on the breed, Threshold 1 (for the identification of LBW kittens) was established at 68% to 113% and Threshold 2 (for the identification of VLBW kittens) at 54% to 82% of the mean birth weight of the breed.

**Figure 4:**
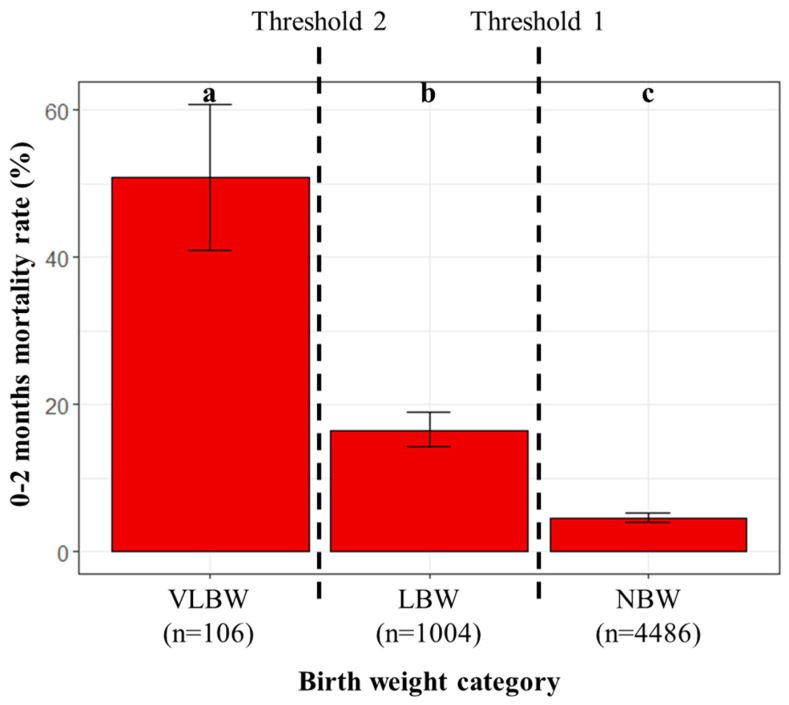
Mortality rate over the first two months of life for normal, low, and very low birth weight kittens (CART analysis; n = 5596 kittens). The error bars represent statistical uncertainty (95% binomial confidence intervals). Mortality rates were significantly different between the three groups (pairwise comparisons by controlling the false discovery rate after a Chi-Square test of independence; different letters at the top of the bars indicate significant differences).

In total, among the 5596 kittens belonging to 15 breeds, 80.2%, 17.9%, and 1.9% of the kittens were normal (NBW), low (LBW), and very low (VLBW) birth weight, respectively, according to our modelling approach. Mortality rates over the first two months were 4.5%, 95% CI [3.9, 5.2], for NBW kittens (203/4486), 16.5%, 95% CI [14.3, 19], for LBW kittens (166/1004), and 50.9%, 95% CI [41, 60.8], for VLBW kittens (54/106; Figure 4).

## 4. Discussion

This work describes a national scale study on feline neonatalogy. The size of the population (1507 litters) was higher than in the previous studies on this topic (15 litters [22], 294 litters [9], 337 litters [23], 694 litters [24], or 1056 litters [12]). Only one study has been conducted on a larger population (7075 litters) [25], but recording was performed at the litter rather than at the individual level, preventing the analysis of parameters such as birth weight. The present work included 15 breeds among the 55 currently recognized by the French feline studbook for purebred cats (LOOF, Pantin, France) [26].

The top-ten breeds owned in France were represented in the study population [27]. Although information was collected on a large sample of kittens (n = 5596), a selection bias could not be excluded, as breeders participated voluntarily. Attentive breeders are probably overrepresented because of their specific interest in birth weight. In addition, birth weights were assessed on site by the breeders, probably under variable measurement conditions (different scales and non-standardized times, from zero to few hours after birth). This is inherent to the nature of the data collection, which was retrospective and multisite.

### 4.1. Postnatal Mortality

In the current study, kitten mortality was assessed between birth and two months of age, the minimum legal age for kitten adoption/sale in France. Its prevalence, 7.6% (95%CI: 6.9–8.3), was similar to that in a previous study in France (7.9%) [25], but lower than in other countries such as the UK (9.1%) [12], Sweden (8.3% between week 1 and 12) [24], or Italy (12%) [23]. Many factors could explain these differences, such as a better motivation of participating breeders in our study, leading to the implementation of more favourable management practices, or breed differences between countries with higher or lower risk of dystocia. Studies at the international level should be conducted to compare the prevalence of 0–2 months kitten mortality in cats and to explore drivers explaining the differences observed (e.g., management practices, genetic lineages, environmental factors).

### 4.2. Mortality Risk Factors at 0–2 Months

The interest in birth weight for better controlling neonatal mortality has been demonstrated in numerous species [8], but studies on birth weight, mortality, and their determinants in cats are scarce. This could be partly related to the limited organisation of the cat breeding world, mainly composed of small breeding facilities with a low rate of professionalization [6,28]. These characteristics, as well as the absence of professional tools for data centralization, make collecting information regarding the first weeks of life of kittens challenging. The present study demonstrated, in a large multibreed population from multiple catteries, the major impact of birth weight on the 0–2 months mortality of purebred kittens. This relationship had already been suggested in an earlier work on a smaller population [9]. A survey conducted in 2019 highlighted that only a quarter of French breeders considered LBW as a risk factor for postnatal mortality but that they did not systematically use weight to identify LBW, sometimes preferring visual observation of newborns (of behaviour or body size) [7].

For the other factors explored, contrary to a previous study [24], litter size was not found to be associated with mortality rate (Table 2). This could be explained by the categorization of litter size (in quartiles), which was chosen to allow the introduction of breed effects on the descriptive model, rather than an introduction as a continuous variable in the model of Ström Holst and Frössling. Contrary to what has been described for piglets [29], litter weight heterogeneity did not seem to impact the mortality rate of kittens. However, litter heterogeneity in cats was about half that in pigs [15]. Interestingly, in dogs, litter heterogeneity close to that described in the current work [30] impacted puppy survival. This difference could be explained by less competition between kittens than between puppies, which is possibly related to the balance between litter size and the number of teats. Indeed, even if there are, in general, two more teats in bitches than in queens (10 vs. 8 [31]), larger litter sizes were reported in dogs than in cats (on average 3 to 8 puppies [32,33] vs. 3 to 5 kittens [22, 23] depending on the breed). Moreover, the number of puppies more frequently exceed the number of teats in dogs compared to cats. In conclusion, the survival of LBW kittens would not be affected by the presence of larger kittens in the litter.

Data was collected through questionnaire. In order to limit memory bias and the length of the questionnaire [34], not all parameters likely to have an impact on postnatal mortality in feline species could be explored. Further research and data collection on other potential risk factors for neonatal mortality described in other species (e.g., parity, maternal age, type of birth, and environmental and managerial conditions in the cattery) may be relevant to improve knowledge of risk factors for postnatal mortality in kittens.

### 4.3. Identification of Birth Weight Thresholds

The main objective of this study was to provide, as in other mammalian species [35], cat breeders and veterinarians with thresholds that would allow them to identify kittens at-risk, as weighing at birth is described as a common practice in catteries [7]. These kittens could then be managed with appropriate care to increase their chances of survival. Moreover, these thresholds would also help breeders to recognize LBW as a health issue. In the current study, two thresholds were determined to identify two groups at increased risk of mortality: Threshold 1 to identify kittens at risk (LBW) and Threshold 2 kittens at high risk (VLBW). Since they were obtained from a feline population born in France between 2000 and 2020, their validation on lineages from other countries is needed.

These thresholds vary depending on the breed (from 60 g to 78 g for Threshold 1 and from 74 g to 104 g for Threshold 2). This variation cannot be explained only by breed variation of birth weight [10,11,12,36] since these thresholds represent a variable proportion of mean birth weight of the breed (Table 3). They also vary between breeds with similar birth weight distributions and equivalent mortality rates. For example, Russian Blue/Nebelung and Egyptian Mau breeds did not differ either in their birth weights or in their mortality rates, but their Thresholds 1 were 92.8 g and 112.6 g, respectively (established at the breed level). Thus, our data show that feline breeds have different sensitivities to birth weight reduction, as described in dogs [30]. More studies are needed to further clarify the relationship between intra-uterine growth restriction and neonatal mortality, including differences between breeds.

Moreover, the population of (very) low birth weight is probably non homogeneous [37,38]: some of them are probably only constitutionally small without having experimented intrauterine growth retardation and, thus, are not particularly at-risk. Further studies are needed to differentiate between pathological LBW and constitutional LBW [38,39], including the exploration of factors leading to the birth of LBW kittens (e.g., maternal nutrition, litter size, intra-uterine position, placental physiology, or underlying pathology). Other newborn parameters proposed in the literature (e.g., biochemical markers [40], morphology [39,41], and vitality score [42]) could help make this distinction.

## 5. Conclusions

Early detection of at-risk kittens is essential to reduce the mortality rate in catteries. This study could help pet professionals (e.g., breeders and veterinarians) build awareness of the issue of LBW. It also provides objective birth weight thresholds, making the identification of at-risk newborns practical. Weighing at birth requires no specific skills but allows for easy identification of kittens that will require care with an inexpensive tool (scale). Beyond the neonatal period, further research is required to study the mid- and long-term consequences of reduced intrauterine growth and low birth weight. In addition, it would be interesting to explore, in depth, the postnatal growth of kittens, the monitoring of which could also be an interesting tool in the field.

## Figures and Tables

**Table 1:** Description of population by breed (n = 5596 kittens from 15 breeds).

Breed	Number of Kittens Included	% of the Total Population	Number of Catteries	Number of Litters	Litters with at Least One Stillborn (%)	Sex Ratio	Mean Birth Weight, Grams (±SD)	Median Litter Heterogeneity, % (IQR)	Mean Litter Size (±SD)	0–2 Months Mortality Rate (%)
Abyssinian/Somali	264	4.7	9	85	5.9	1.3	97.2 (±11.8)	8.2 (4.3–10.7)	3.2 (±1.2)	7.6 (4.7–11.5)
Balinese/Mandarin/Oriental/Siamese	138	2.5	9	35	11.4	1.4	95.4 (±13.1)	8 (6.7–10.4)	4.3 (±2.1)	4.3 (1.6–9.2)
Bengal	206	3.7	11	57	10.5	1.1	88.2 (±15.7)	10.2 (6.4–14.4)	4.3 (±1.6)	10.2 (6.4–15.2)
Birman	607	10.8	30	196	8.7	0.9	95.8 (±14.7)	7.7 (5.3–9.9)	3.5 (±1.2)	5.6 (3.9–7.7)
British	810	14.5	23	216	19	1.1	98.4 (±17.1)	8.5 (5.2–11.9)	3.9 (±1.4)	9.8 (7.8–12)
Chartreux	274	4.9	9	69	2.9	1.1	110.4 (±18.5)	7.5 (4.6–10.7)	4.1 (±1.3)	4.7 (2.6–8)
Egyptian Mau	122	2.2	6	29	31	1.2	92.3 (±21.2)	10 (7.9–13.7)	4.2 (±1.3)	12.3 (7–19.5)
Maine Coon	892	15.9	39	217	16.6	1.3	119.1 (±18.7)	8.3 (5.6–11.8)	4.3 (±1.9)	7.4 (5.8–9.3)
Norwegian Forest	806	14.4	17	199	10.1	1.1	109.9 (±17.7)	7.5 (5.3–10.8)	4.2 (±1.5)	4.7 (3.4–6.4)
Persian/Exotic	365	6.5	22	128	16.4	1.1	85.5 (±15)	7.3 (4.5–12.1)	3.1 (±1.1)	14 (10.6–18)
Ragdoll	331	5.9	8	78	15.4	1.1	100.3 (±13.5)	8.2 (5.9–11.2)	4.3 (±1.5)	2.4 (1–4.7)
Russian Blue/Nebelung	108	1.9	4	25	8	1.3	92.7 (±15.2)	6 (4.7–9.4)	4.4 (±1.7)	13.9 (8–21.9)
Scottish/Highland	133	2.4	11	33	21.2	0.9	89.5 (±12.7)	9.6 (6.4–12.1)	4.2 (±1.2)	11.3 (6.5–17.9)
Siberian	419	7.5	15	105	20	1.3	99.3 (±16.7)	8.6 (4.8–13)	4.1 (±1.6)	8.8 (6.3–12)
Sphynx	121	2.2	11	35	20	0.8	90.3 (±14.6)	8.2 (6.2–12.3)	3.4 (±1.7)	4.1 (1.4–9.4)
Total	5596	100	194	1507	13.9	1.1	101.9 (±19.4)	8.1 (5.3–11.6)	3.9 (±1.6)	7.6 (6.9–8.3)

SD = standard deviation; IQR = interquartile range.

**Table 2:** Predictive factors for 0–2 months mortality (n = 5596 kittens, generalised linear mixed-model). Birth weight, litter size, and litter heterogeneity categories were constructed based on quartile values calculated at the breed level.

Factors	*p*-Value	Odds Ratio [95%CI]
Season of birth		
Autumn	0.055	1.65 [0.99, 2.75]
Spring		1 (Ref.)
Summer	0.015	1.73 [1.11, 2.69]
Winter	0.506	1.26 [0.64, 2.51]
Presence of stillborn in the litter		
No		1 (Ref.)
Yes	0.403	1.23 [0.76, 1.99]
Litter size		
Q1	0.094	1.61 [0.92, 2.83]
Q2		1 (Ref.)
Q3	0.108	1.75 [0.88, 3.45]
Q4	0.040	1.95 [1.03, 3.68]
Birth weight		
Q1	<0.001	10.16 [5.39, 19.12]
Q2	<0.001	4.76 [2.57, 8.79]
Q3	0.005	2.43 [1.30, 4.55]
Q4		1 (Ref.)
Sex		
Female		1 (Ref.)
Male	0.001	1.73 [1.27, 2.37]
Litter heterogeneity		
Q1	0.197	1.44 [0.83, 2.49]
Q2		1 (Ref.)
Q3	0.405	1.26 [0.73, 2.16]
Q4	0.057	1.70 [0.98, 2.92]

**Table 3:** Birth weight thresholds for the identification of kittens at significantly higher risk of 0–2 months mortality for 15 feline breeds.

Group	Mean BW, Grams	Threshold 1 (Identification of LBW Kittens)		Threshold 2 (Identification of VLBW Kittens)	
		In Grams	% of Mean BW	In Grams	% of Mean BW
Abyssinian/Somali	97.2	94	96.7	60 *	61.7
Balinese/Mandarin/Oriental/Siamese	95.4	82 *	85.9	78	81.8
Bengal	88.2	84	95.2	60 *	68.0
Birman	95.8	74	77.3	60 *	62.7
British	98.4	87	88.4	61	62.0
Chartreux	110.4	100	90.6	60 *	54.3
Egyptian Mau	92.3	104	112.6	61	66.1
Maine Coon	119.1	81	68.0	75	63.0
Norwegian Forest	109.9	94	85.5	60 *	54.6
Persian/Exotic	85.5	82	95.9	60 *	70.2
Ragdoll	100.3	84	83.8	60 *	59.8
Russian Blue/Nebelung	92.7	86	92.8	60 *	64.7
Scottish/Highland	89.5	77	86.0	60 *	67.0
Siberian	99.3	90	90.7	63	63.5
Sphynx	90.3	76	84.2	60 *	66.5

* Threshold established at the species level. BW: birth weight; LBW: low birth weight; VLBW: very low birth weight.

## Data Availability

Data are available from the corresponding author on reasonable request.
